# Compressed-sensing accelerated 4D flow MRI of cerebrospinal fluid dynamics

**DOI:** 10.1186/s12987-020-00206-3

**Published:** 2020-07-16

**Authors:** Elena Jaeger, Kristina Sonnabend, Frank Schaarschmidt, David Maintz, Kilian Weiss, Alexander C. Bunck

**Affiliations:** 1 Department of Diagnostic and Interventional Radiology, University of Cologne, Faculty of Medicine and University Hospital Cologne, Kerpener Street 62, 50937 Cologne, Germany; 2 Philips GmbH, Hamburg, Germany; 3 Institute of Cell Biology and Biophysics, Biostatistics Department, Leibniz University Hannover, Hannover, Germany

**Keywords:** Compressed sensing, 4D flow MRI, Cerebrospinal fluid, CSF

## Abstract

**Background:**

4D flow magnetic resonance imaging (MRI) of CSF can make an important contribution to the understanding of hydrodynamic changes in various neurological diseases but remains limited in clinical application due to long acquisition times. The aim of this study was to evaluate the accuracy of compressed SENSE accelerated MRI measurements of the spinal CSF flow.

**Methods:**

In 20 healthy subjects 4D flow MRI of the CSF in the cervical spine was acquired using compressed sensitivity encoding [CSE, a combination of compressed sensing and parallel imaging (SENSE) provided by the manufacturer] with acceleration factors between 4 and 10. A conventional scan using SENSE was used as reference. Extracted parameters were peak velocity, absolute net flow, forward flow and backward flow. Bland–Altman analysis was performed to determine the scan-rescan reproducibility and the agreement between SENSE and compressed SENSE. Additionally, a time accumulated flow error was calculated. In one additional subject flow of the spinal canal at the level of the entire spinal cord was assessed.

**Results:**

Averaged acquisition times were 10:21 min (SENSE), 9:31 min (CSE4), 6:25 min (CSE6), 4:53 min (CSE8) and 3:51 min (CSE10). Acquisition of the CSF flow surrounding the entire spinal cord took 14:40 min. Bland–Altman analysis showed good agreement for peak velocity, but slight overestimations for absolute net flow, forward flow and backward flow (< 1 ml/min) in CSE4–8. Results of the accumulated flow error were similar for CSE4 to CSE8.

**Conclusion:**

A quantitative analysis of acceleration factors CSE4–10 showed that CSE with an acceleration factor up to 6 is feasible. This allows a scan time reduction of 40% and enables the acquisition and analysis of the CSF flow dynamics surrounding the entire spinal cord within a clinically acceptable scan time.

## Background

Changes in the cerebrospinal fluid (CSF) flow dynamics have been found to be associated with different neurological diseases such as hydrocephalus [1], Chiari malformation [2, 3], syringomyelia [4] and Alzheimer disease [5]. The quantification of the CSF flow is necessary for a better understanding of the physiology and pathophysiology of CSF dynamics and may assist in diagnosis and treatment guidance of CSF related diseases. At present, two-dimensional phase-contrast magnetic resonance imaging (2D flow MRI) is the most common technique to quantify the CSF flow non-invasively [6]. Since this method is two-dimensional it is not sufficient to fully represent the complex physiological or pathological flow dynamics of the CSF including multidirectional flow occurring in patients with Chiari malformation, inside of arachnoid cysts and syringes as well as during reflux from the third into the lateral ventricles [7, 8]. To represent complex flow patterns adequately a time-resolved three-dimensional MRI measurement (4D flow MRI) method is necessary [9, 10]. However, due to the large amount of data that needs to be recorded, the acquisition of a 4D flow MRI data set is accompanied with long scan times and therefore, rarely integrated into clinical routine scan protocols.

To accelerate 4D flow MRI acquisitions, different methods were proposed by exploiting spatial–temporal correlations or using compressed sensing. However, most work focused on cardio-vascular applications [11–15] and no work exists on accelerated 4D flow MRI of the CSF.

The concept of compressed sensing is a promising way to accelerate MRI measurements. It was first proposed in 2006 by Donoho [16] and transferred to MRI by Lustig et al. [17]. Compressed sensing can reduce the required amount of data by random undersampling of the k-space, if an image can be assumed to be “sparse” in a suitable transform domain [18]. Further image acceleration can be accomplished by combining compressed sensing and parallel imaging [18, 19], herein referred to as compressed SENSE (CSE) [20, 21]. Previous studies have shown that CSE can accelerate scan times while obtaining an acceptable quality in aortic [13, 22, 23] and carotid [24] blood flow using acceleration factors between R = 3 and R = 7. In comparison to blood flow, velocities of the CSF are low and therefore more difficult to measure. Contrast of the tissue is lower and no contrast agent can be used making an exact acquisition more challenging. In addition, when measuring the blood flow in the chest or abdomen more coils can be used which improves quality.

The aim of this study was to investigate the applicability of the combination of compressed sensing and parallel imaging to the quantification of CSF flow using 4D flow MRI to improve its clinical applicability. To estimate the maximum feasible image acceleration using CSE, different CSE factors were compared to the standard parallel imaging acceleration using SENSE regarding image quality and different flow parameters, such as peak velocity, forward volume flow, backward volume flow and absolute net flow in healthy subjects. Finally, to further investigate the potential of the CSE acceleration the proposed method was applied to measure the CSF flow surrounding the entire spinal cord (C1-L1) in one healthy subject by the combination of 2 separate acquisitions.

## Methods and material

### Study population

20 healthy subjects (11 women, 9 men, mean age ± SD: 29.7 ± 13.04 years) with no history of neurological or spinal diseases were recruited. The study was approved by the institutional ethics committee and informed consent was obtained from all subjects prior to the study.

### Image acquisition

All examinations were obtained using a clinical 3 T MRI system (Ingenia; Philips Healthcare, Best, The Netherlands). Data were acquired using a spoiled gradient echo sequence with a 16-channel head and neck coil array and the built‐in 8-channel posterior coil array. During the examination, the peripheral heart rate was recorded using a wireless pulse oximeter to allow for retrospective synchronization of the data. Beforehand, a survey scan and a sagittal 3D T2-weighted scan were performed for planning purposes. The imaging volume of the 4D flow acquisition was chosen to cover the cerebral aqueduct and the cervical spine (C1-C7). Each volunteer underwent one scan using SENSE with an acceleration factor of R = 3.75. and four scans using CSE (acceleration factors R = 4, 6, 8 and 10—termed as CSE4, CSE6, CSE8 and CSE10). The CSE sequence is a combination of SENSE and compressed sensing techniques. Both sequences used in this work were provided by the manufacturer as product sequences (Compressed SENSE/ SENSE, Philips Healthcare). The acquired trajectory was cartesian. A pseudorandom undersampling pattern with fully sampling of the center and a randomized pattern of the remaining k-space was used. No sparsity was used in the time domain. A regularized L1-iterative norm in combination with wavelet transform as sparsifying transform was used for reconstruction of the images. In 15 out of the 20 subjects a second scan using SENSE was performed to evaluate the scan-rescan reproducibility. To mitigate the bias from ordering effects the acquisitions were performed in random order, except for the second scan using SENSE, which was always acquired at the end of the session. Repeated scans were acquired without repositioning of the subjects. The MRI protocol took about 60 min depending on the subjects’ heart rate. All imaging parameters are shown in Table 1. The FOV was resized, if necessary.

**Table 1:** Acquisition parameters

Parameter	Cervical spinal canal	C1-L1
Repetition time (ms)	8.2	8.3
Echo time (ms)	5.1	4.0
Field of view (mm^3^)	[220–240] × [240–250] × 30 (FH × AP × RL)	277.6 × 277.6 × 30 (FH × AP × RL)
Acquisition voxel size (mm^3^)	1.2 × 1.2 × 1.2	1.5 × 1.5 × 1.5
Reconstructed voxel size (mm^3^)	0.6 × 0.6 × 0.6	0.88 × 0.88 × 0.75
Acquired temporal resolution (ms)	65.6	66.4
VENC(cm/s) (all spatial directions)	15	15
Flip angle (deg)	4	4
Reconstructed cardiac phases	15	15
Acceleration factor	3.75,4,6,8,10	6

In one additional healthy subject an acquisition of the cervical and the thoracic spinal canal (C1-L1) was performed by splitting the FOV in two overlapping sagittal stacks using an acceleration factor of R = 6 (CSE6). To reduce the effect of heart rate variability between the two stacks 15 cardiac phases were reconstructed. Scan parameters are also listed in Table 1.

Reconstruction was performed on-line using standard imaging reconstructing hardware (32 GB RAM, Intel Xeon E5-1620 CPU). Concomitant gradient fields were corrected on-line. Eddy current induced background phases were corrected during post processing.

### Image analysis

**Fig. 1:**
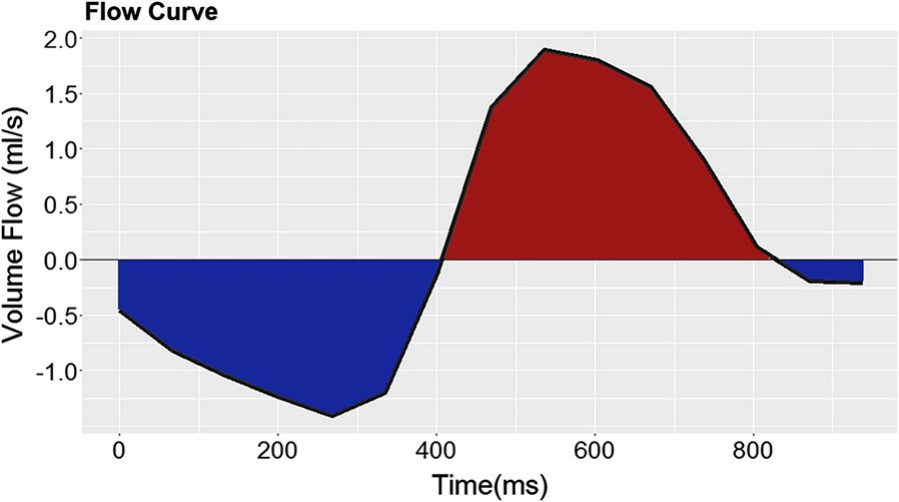
CSF flow curve. Exemplary flow curve at the level of C1. Data were extracted from one SENSE acquisition of one subject. Red indicates forward flow, blue indicates backward flow

The velocity mapping data were analyzed using the commercially available GTFlow software (version 3.1, Gyrotools LLC, Winterthur, Switzerland). Built-in correction of eddy currents induced background phases were applied and by default, cranially directed velocities were defined as positive. For each volunteer 7 regions of interest (ROI) were defined in the scan using SENSE located at the center of the cervical vertebrae (C1–C7). In each ROI the subarachnoid space of the spinal cord was manually outlined. Contours were drawn at the time point with the most apparent flow and copied to all time points. The defined contours were transferred to the scan using CSE and the repeated scan using SENSE and shifted manually, if necessary. To provide good agreement the spinal canal and the vertebral vessels served as anatomical landmarks. Flow curves were automatically extracted by GTFlow as the sum of the pixels of the contour. Forward volume flow (FF) was calculated as the sum of the cranially directed flow, backward volume flow (BF) was calculated as the sum of the caudally directed flow (see Fig. 1). Additionally, the absolute net flow (AF) was calculated as the average of absolute forward and backward volume flow. Peak velocity (PV) was extracted from the pixel with the maximum velocity of all time points within the ROI.

Data covering the C1-L1 region were processed equally. ROIs were defined at the level of the midportion of each vertebra and additionally at each intervertebral disc, resulting in 36 analyzed ROIs. Both stacks were visually overlapped using Matlab (Matlab 2019a, The MathWorks, Inc., Natick, MA) and flow patterns were visualized by generating pathlines.

### Statistical analysis

For statistical analysis, the open source software package R-studio (version 3.6.2) was used. Bland–Altman analyses were performed to assess the agreement and the correlation between the scans using SENSE and the scans using different CSE factors as well as scan-rescan reproducibility between repeated scans using SENSE. Limits of agreement were corrected as recommended for repeated measurements using REML [25].

To additionally assess the significance and magnitude of the potential bias of CSE methods compared to SENSE, pair differences between CSE methods and SENSE were calculated as SENSE-SENSE2 and SENSE-CSE for each position along the spinal cord within each patient. This approach accounts for the dependency of the 7 acquired slices within the subjects. These pair differences were analyzed in a linear mixed effect model, with the mean pair difference as fixed effect and between patient variance as random effect. Based on the fitted models we computed: (1) *p* values for the test whether mean pair difference is equal to 0 (null hypothesis, no bias compared to SENSE) or differs from 0 (alternative hypothesis, CS methods biased compared to SENSE), and (2) 95% confidence limits for the mean pair difference.

Additionally, the accumulated flow error E_R_ as proposed by Giese et al. [26] was calculated (Eq. 1). This parameter is an approach that is more sensitive to detect possible temporal differences between the flow curves. Since the net CSF flow should be close to zero, instead of dividing by the sum of the net flow it was divided by the sum of the absolute net flow.

1 $${E}_{R}=\frac{1}{{n}_{S}}\sum_{S=1}^{{n}_{S}}\left(\frac{1}{{n}_{C}}\sum_{C=1}^{{n}_{C}}\left(\frac{\sum_{t=0}^{{t}_{{n}_{T}}}\left|{{Q}_{\text{SENSE}}}_{S,C}\left(t\right)-{{Q}_{\text{CSE R}}}_{S,C}\left(t\right)\right|}{\sum_{t=0}^{{t}_{{n}_{T}}}{{|Q}_{\text{SENSE}}}_{S,C}\left(t\right)|}\right)\right)$$

${n}_{S}$ corresponds to the number of subjects, ${n}_{C}$ to the number of contours and ${n}_{T}$ to the number of timepoints. ${{Q}_{\text{SENSE}}}_{S,C}$ and ${{Q}_{\text{CSE R}}}_{S,C}$ delineates the flow through the contour C in the subject S over time in the scan using SENSE and CSE.

## Results

Scans were completed in all subjects, none of the scans had to be aborted or repeated. In one subject all slices of the second scan using SENSE and in another subject slices from C3–C7 of the scans using SENSE and CSE6 were excluded due to motion artifacts. Acquisition times averaged over all subjects were 10:21± 1:19 min (SENSE), 9:31 ± 1:12 min (CSE4), 6:25 ± 0:51 min (CSE6), 4:53 ± 0:38 min (CSE8) and 3:51 ± 0:29 min (CSE10). The average reconstruction time of CSE accelerated acquisitions took around 10 min per scan.

**Fig. 2:**
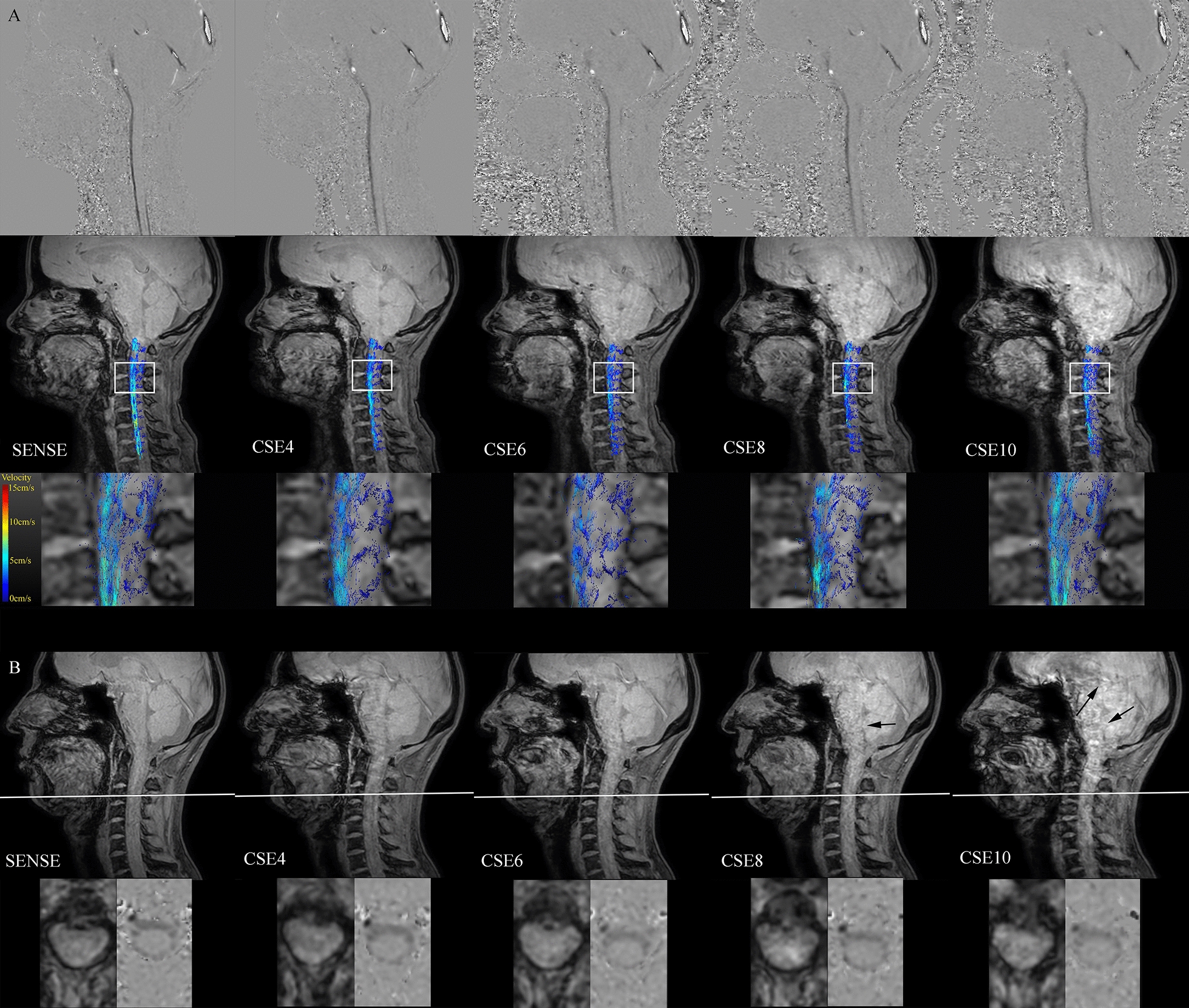
Magnitude, phase contrast and pathline images. **a** Phase images (top) with feet-head encoded velocity at peak flow (white = caudal flow, black = cranial flow), magnitude with pathlines (middle) and zoomed-in pathlines (bottom). Acceleration factors labeled correspondingly. Pathline screenshots were taken at 734 ms (SENSE), 640 ms (CSE4), 795 ms (CSE6), 810 ms (CSE8), and at 763 ms (CSE10). For better visualization of the pathlines, additional contours were defined at each intervertebral disk. **b** Magnitude images of one subject (top) with one exemplary ROI. Axial magnitude and phase images of the corresponding ROI in one examplary slice (white line) (bottom). Acceleration factors labeled correspondingly. Pathline screenshots were taken at 620 ms (SENSE), 625 ms (CSE4), 622 ms (CSE6), 620 ms (CSE8), 625 ms (CSE10). Imaging artifacts are highlighted with black arrows in CSE8 and CSE10

Figure 2a exemplary shows phase-contrast, magnitude and pathline images of one subject. Figure 2b shows resulting magnitude and phase-contrast images with the corresponding ROI. A decrease in quality with increasing CSE acceleration factor can be noticed. Time resolved animations of the pathlines are available in the supplementary material (see Additional files 1, 2,  3,  4, 5).

**Fig. 3:**
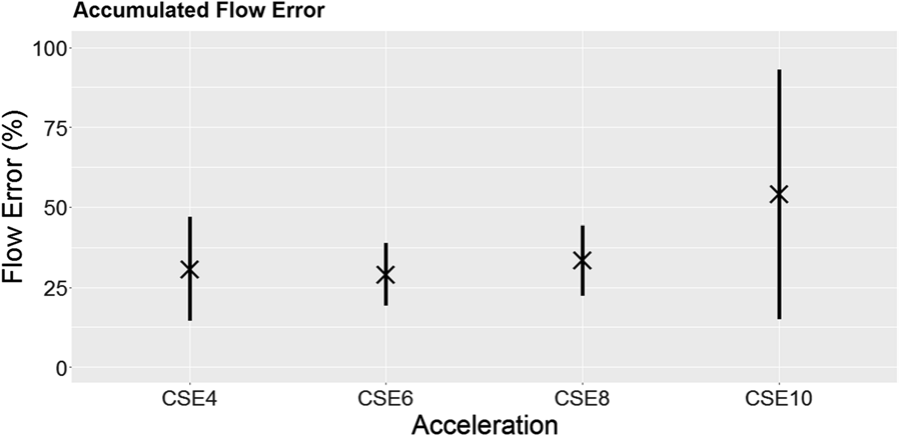
Time accumulated flow error. Time accumulated flow error of all CSE acceleration factors. Error bars represent the standard deviation over all subjects

Figure 3 shows the time-accumulated flow error averaged over all subjects. While CSE4, CSE6 and CSE8 show comparable flow errors around 30%, the tendency of an increasing error with increasing acceleration can still be observed. The largest deviation can be seen in CSE10 with a flow error of 53.9 ± 39.1%.

**Fig. 4:**
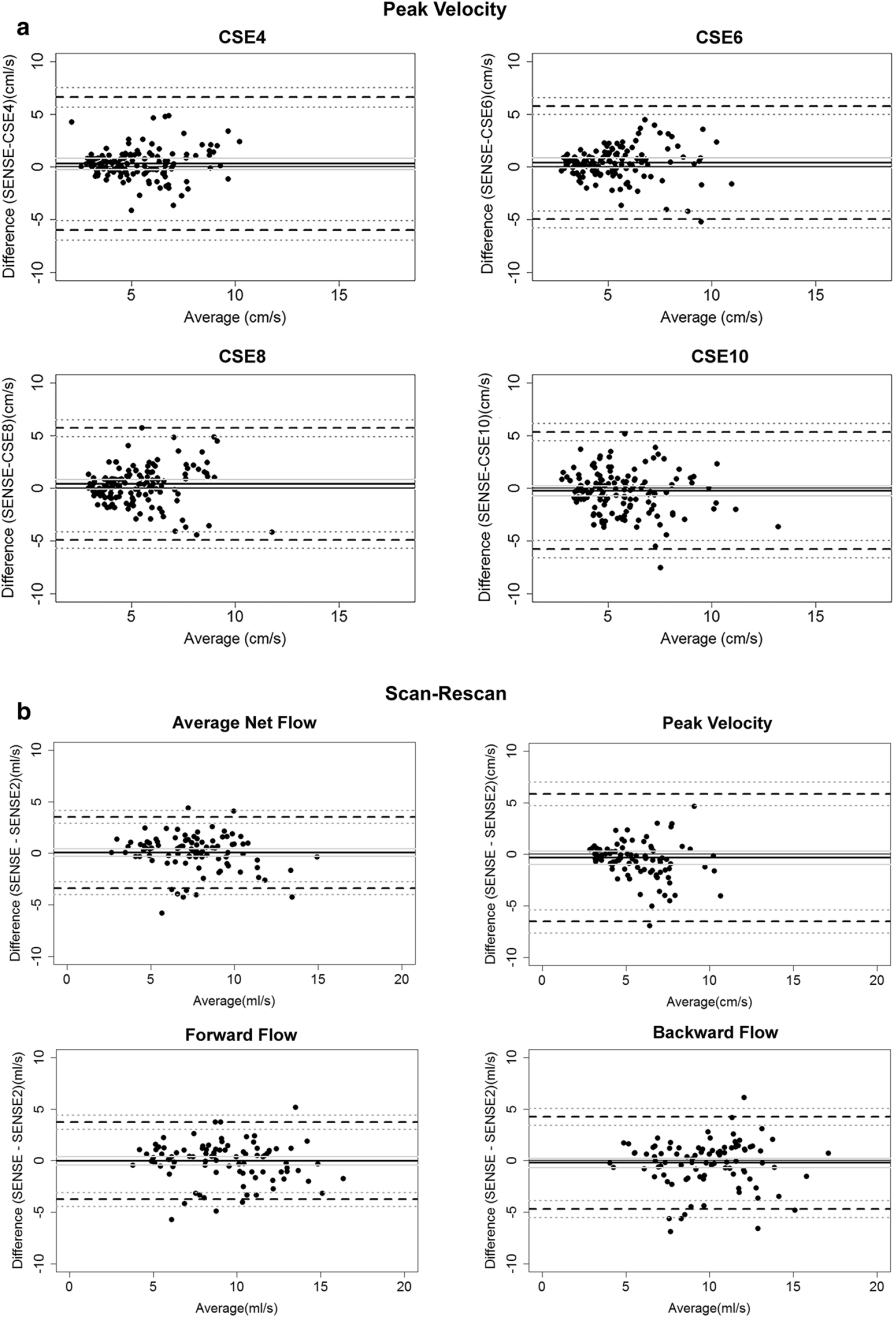
Bland–Altman analysis of peak velocity and scan-rescan agreement. **a** Bland–Altman plots comparing SENSE and CSE in 140 measurements for CSE4, 8 and 10 and 135 for CSE6 in 20 subjects over 7 contours. CSE acceleration factor labeled correspondingly. Solid lines indicate the mean difference with the corresponding 95% confidence interval, outer dashed lines indicate the limits of agreement with the corresponding 95% confidence interval. **b** Bland–Altman plots for scan-rescan agreement of 93 measurements. Analyzed flow parameter labeled correspondingly. Solid lines indicate the mean difference with the corresponding 95% confidence interval; outer dashed lines indicate the limits of agreement with the corresponding 95% confidence interval

Results of the Bland–Altman analyses are shown in Table 2. For Bland–Altman plots see Fig. 4 and Additional files 6,  7,  8.

**Table 2:** Results of Bland–Altman analysis for all flow parameters

Flow parameter	Acceleration factor	MD (CI)	SD	UL (CI)	LL (CI)	Absolute mean
Absolute net flow (ml/s)	CSE4	0.31 (0.1; 0.5)	1.19	2.65 (2.3; 3.0)	− 2.02 (− 2.4; − 1.7)	7.94
	CSE6	0.82 (0.6; 1.1)	1.42	3.60 (3.2; 4.0)	− 1.96 (− 2.4; − 1.5)	7.45
	CSE8	1.13 (0.9; 1.4)	1.4	3.88 (3.5; 4.3)	− 1.61 (− 2.0; − 1.2)	7.12
	CSE10	0.82 (0.5;1.2)	2.11	4.95 (4.3; 5.6)	− 3–30 (− 3.9; − 2.7)	7.43
	SENSE2	0.08 (− 0.3; 0.4)	1.77	3.55 (3.0; 4.2)	− 3.39 (− 4.0; − 2.8)	7.59
	SENSE					8.25
Peak velocity (cm/s)	CSE4	0.31 (− 0.2; 0.8)	3.22	6.63 (5.7; 7.6)	− 6.0 (− 5.1; − 6.9)	5.31
	CSE6	0.41 (− 0.04;0.9)	2.74	5.78 (5.0; 6.6)	− 4.96 (− 4.2; − 5.8)	5.21
	CSE8	0.40 (− 0.05;0.9)	2.71	5.72 (5.0; 6.5)	− 4.91 (− 4.1; − 5.7)	5.20
	CSE10	− 0.22 (− 0.7;0.2)	2.84	5.33 (4.5; 6.1)	− 5.79 (− 5.0; − 6–6)	5.85
	SENSE2	− 0.32 (− 1.0; 0.3)	3.16	5.88 (4.8; 7.0)	− 6.52 (− 5.4; − 7.7)	5.92
	SENSE					5.598
Forward flow (ml/s)	CSE4	0.78 (0.5; 1.02)	1.44	3.61 (3.6; 5.6)	− 2.05 (− 1.6; − 2.5)	8.93
	CSE6	0.96 (0.7; 1.2)	1.60	4.10 (3.6; 4.6)	− 2.18 (− 1.7; − 2.6)	8.76
	CSE8	0.90 (0.6; 1.2)	1.87	4.56 (4.0; 5.1)	− 2.77 (− 2.2; − 3.3)	8.81
	CSE10	0.31 (− 0.4; 1)	3.91	7.97 (6.8; 9.1)	− 7.35 (− 6.2; − 8.5)	9.40
	SENSE2	− 0.003 (− 0.4; 0.4)	1.91	3.76 (3.1; 4.4)	− 3.76 (− 3.1; − 4.5)	9.23
	SENSE					9.71
Backward flow (ml/s)	CSE4	0.33 (0.08; 0.6)	1.48	3.23 (2.8; 3.7)	− 2.58 (− 2.1; − 3.0)	9.77
	CSE6	0.65 (0.3; 0.97)	1.90	4.37 (3.8; 4.9)	− 3.06 (− 2.5; − 3.6)	9.49
	CSE8	0.87 (0.5; 1.2)	2.02	4.83 (4.3; 5.4)	− 3.10 (− 2.5; − 3.7)	9.23
	CSE10	0.57 (− 0.08; 1.2)	3.90	8.21 (7.1; 9.3)	− 7.07 (− 5.9; − 8.2)	9.53
	SENSE2	− 0.21 (− 0.7; 0.3)	2.29	4.28 (3.5; 5.1)	− 4.71 (− 3.9; − 5.5)	9.97
	SENSE					10.1

*SD* standard deviation, *CI* confidence interval, *MD* mean difference, *UL* upper limit, *LL* lower limit

Upper limit of agreement represents the mean difference + 1.96 standard deviations, lower limit of agreement represents the mean difference − 1.96 standard deviations. All numbers in parentheses indicate the 95% confidence interval

SD is given for difference between CSE and SENSE. Deviations between CSE and SENSE were evaluated in 140 measurements for CSE4, 8 and 10 and 135 for CSE6. Deviations between SENSE 2 and SENSE were evaluated for 93 measurements. Absolute mean values are given for reference

Limits of agreement (LoA) of CSE4, CSE6 and CSE8 were similar for AF, FF and BF with a slight increase with increasing acceleration factors. In CSE10 a notable widening of the confidence intervals can be observed for all parameters. The PV showed relatively larger confidence intervals than other parameters in all acceleration factors with LoAs between 10–12 cm/s, although the mean differences of PV showed only minor deviations in all acceleration factor (CSE4: 0.3 cm/s; CSE6: 0.43 cm/s; CSE8: 0.33 cm/s; CSE10: − 0.223 cm/s). Confidence intervals (CI) of PV included zero in all acceleration factors. The mean differences in AF and BF were smallest in CSE4 (AF: 0.3 ml/s and BF: 0.3 ml/s), increased in CSE6 (AF:0.8 ml/s; BF: 0.6 ml/s) and were largest in CSE8 (1.1 ml/s and BF: 0.9 ml/s).

Table 3 shows the resulting mean pair differences.

**Table 3:** Statistical interference for the pair differences of CSE vs. SENSE and SENSE2 vs. SENSE

Flow parameter	Acceleration-method	Mean pair difference	Lower CI	Upper CI	*p* value	Sign
Average net flow (ml/s)	SENSE2	0.08	− 0.71	0.87	0.84	
	CSE4	0.31	− 0.05	0.67	0.10	
	CSE6	0.82	0.37	1.27	0.002	**
	CSE8	1.13	0.82	1.44	5.59∙10^–7^	***
	CSE10	0.82	0.15	1.49	0.02	*
Peak velocity (cm/s)	SENSE	− 0.32	− 1.24	0.60	0.49	
	CSE4	0.31	− 0.22	0.85	0.26	
	CSE6	0.41	− 1.18	1.00	0.18	
	CSE8	0.40	− 0.21	1.00	0.20	
	CSE10	− 0.23	− 0.72	0.26	0.36	
Forward flow (ml/s)	SENSE2	− 0.003	− 0.84	0.84	0.99	
	CSE4	0.78	0.29	1.28	0.01	**
	CSE6	0.96	0.42	1.50	0.002	**
	CSE8	0.90	0.33	1.46	0.005	**
	CSE10	0.31	− 0.47	1.09	0.44	
Backward flow (ml/s)	SENSE2	− 0.21	− 1.22	0.79	0.67	
	CSE4	0.33	− 0.11	0.77	0.15	
	CSE6	0.65	0.02	1.28	0.05	
	CSE8	0.87	0.32	1.42	0.01	**
	CSE10	0.57	− 0.19	1.33	0.15	

*Lower CI* lower confidence interval, *Upper CI* upper confidence interval, *p* value *p* value for the test whether mean pair difference is equal to 0 (null hypothesis) or differs from 0 (alternative)

Interference for mean pair difference for SENSE–SENSE2 and SENSE–CSE, based on mixed model fits

Stars indicate that the mean pair difference is significantly different from 0 (* 0.05 > *p* > 0.01; ** 0.01 > *p* > 0.001; *** 0.001 > *p*)

There were no significant differences for PV for all acceleration factors. CSE4, 6 and 8 showed a significant difference for FF of 0.78 ml/s (CSE4), 0.96 ml/s (CSE6) and 0.90 ml/s (CSE8). BF had no significant differences except for CSE8 with an estimated difference of 0.87 ml/s. There was a significant difference for AF for CSE6, 8 and 10, with CSE8 showing the highest difference of 1.13 ml/s (p-value 5.59∙10^–7^). The significant difference for CSE6 and 10 was 0.82 ml/s with CIs of 0.37–1.27 ml/s (CSE6) and 0.15–1.49 ml/s (CSE10). There is a tendency to underestimate values in all CSE acceleration factors.

The test–retest comparison (see Fig. 4b) showed small differences between the first and the second scan using SENSE in all parameters (mean differences: AF: 0.08 ml/s; PV: − 0.32 ml/s; FF: − 0.003 ml/s; BF: − 0.215 ml/s). CIs included 0 in all parameters. In all parameters the LoA for repeated scans using SENSE were comparable to the LoA in CSE8. The standard deviation (SD) of repeated scans using SENSE acquisitions was higher than in scans using CSE4, CSE6 and CSE8. Mean pair difference showed no significant differences between the first and the second scan using SENSE in all parameters assessed.

**Fig. 5:**
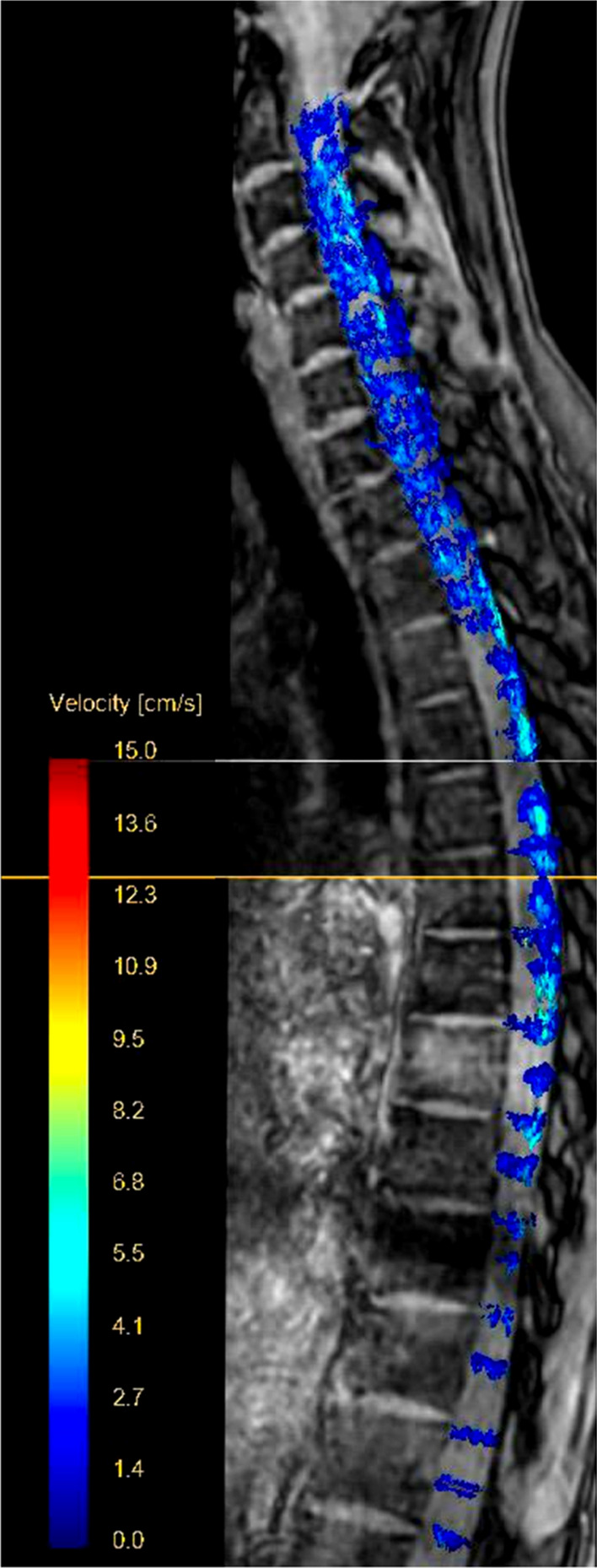
Pathline Screenshot. 4D flow of spinal cord (C1-L1). Magnitude image with pathlines in one healthy subject

Data covering the C1-L1 region were acquired successfully. The acquisition time took 7:20 min per stack and 14:40 min in total. Time-resolved animations of recorded pathlines are available in the supplementary material (see Additional file 9). A screenshot of the pathlines is shown in Fig. 5.

## Discussion

To our knowledge, this is the first study evaluating the feasibility of CSE acceleration for 4D flow MRI of CSF flow dynamics. Moreover, by applying an appropriate CSE acceleration, we were able to demonstrate that it is feasible to measure the CSF flow surrounding the entire spinal cord during a single examination with a clinically acceptable scan time.

Peak flow velocities (5.6 ± 2.7 cm/s) were in good agreement with results of previous studies by Pahlavian et al. [27] with a peak velocity of 6.0 ± 1.7 cm/s, by Bunck et al. [9] with values between 3.0 and 4.8 cm/s and by Lindstrøm et al. [28] with values between 1.8 and 4.2 cm/s.

The flow parameters analyzed in this study showed a good test–retest agreement between repeated scans using SENSE. The SD of 3.16 cm/s for PV and around 2 ml/s for BF, AF and FF seem high compared to the CSE data. This can be attributed to both the smaller number of subjects in the test–retest group naturally leading to wider CIs and the physiological fluctuations due to the delay between the acquisitions of the first and the second scan using SENSE. To minimize physiological differences, like respiration or varying heart rate, the order of acquisitions was randomized except for the second SENSE accelerated scan. This may explain an increased SD of the second scan using SENSE compared to the scans using CSE4 and CSE6. Additionally, reduced movement and physiological variations due to the shorter scan time in scans using CSE4 and CSE6 may lead to increased accuracy. This could also serve as a possible explanation why CSE10 showed no significant differences for FF, BF and PV.

Overall Bland–Altman analyses showed good agreement between scans using CSE and SENSE with acceleration factors up to CSE8. The LoA and the SD of the PV are relatively wider than for the rest of the parameters. This finding can most likely be attributed to the internal process of determining this specific value. The PV is defined as the pixel with the highest velocity. Therefore, this parameter can be subject to large fluctuations due to noise. Another reason that could contribute to deviations is the temporal resolution and the temporal smoothing by 4D flow PCMRI [29, 30]. This finding was also observed in the ventricular structures by Stadlbauer et al. [10] and in the cerebral aqueduct by Sartoretti et al. [31]. Although in all parameters the mean difference of CSE10 is comparable to CSE4 and CSE6 and showed no significant difference for FF, BF, and PV, wide confidence intervals, a high accumulated flow error and a substantially decreased image quality led to the conclusion that CSE10 is beyond adequate acceleration factors. SD of the scan-rescan measurement was between 1.77 and 2.29 ml/s. There were no significant differences for CSE4 for BF and AF and for CSE6 for BF. The significant differences that were detected for CSE4 and CSE6 are small (MD < 0.5 ml/s for CSE4, MD < 0.9 ml/s for CSE6). Those deviations are probably clinically not relevant as the alterations in flow velocities such as in stenotic flow are usually well beyond these minor deviations [2]. Small CIs indicate a higher accuracy due to lower variations within different subjects.

Results of the accumulated flow error were broadly in line with results of the Bland–Altman analyses. A flow error around 30% is higher than reported for cardiovascular 4D flow MRI, for which errors between 15 and 20% in aortic flow and an error between 20–30% in the pulmonary arteries have been described [11, 26]. This can most likely be attributed to the CSF flow being more sensitive to disturbances and the smaller flow velocities making stronger gradients necessary [8]. Additionally, CSF velocities are close to zero in a large portion of the cardiac cycle, which increases the measurement error when using a PCMRI sequence with single encoding velocity. Also, outlining of the spinal CSF can be challenging, e.g. by the contamination of adjacent blood vessels [9], especially because we chose to shift the contours manually and not automatically. Previous studies have shown that respiration also effects the CSF flow in both direction and speed [32–34]. This might be an additional cause for larger errors between repeated scans. Although CSE6 and CSE8 showed similar results in the Bland–Altman analysis and the accumulated flow error, defining contours in images acquired with CSE8 may be difficult due to the reduced magnitude image quality (see Fig. 2). This could potentially lead to greater deviations than observed in our study, as all contours were outlined in the SENSE accelerated scan and also needs to be considered when choosing the appropriate acceleration factor. Moreover, in contrast to CSE8, mean deviations were less than 10% of absolute values for *all* tested parameters when applying CSE6 acceleration. Additionally, CSE8 showed a significant difference for AF, FF and BF with a mean difference > 1 ml/s for AF whereas CSE6 only showed a significant difference for AF and FF with mean differences always < 1 ml/s. Compared to SENSE and CSE4, the CSE6 scan achieved a noticeable reduction in scan time. It is possible that the reduced scan time is the reason for the lower accumulated flow error and may improve image quality by reduced motion artifacts. It would also be possible to make use of the time gained in CSE6 by e.g. choosing a higher temporal resolution. Accordingly, we regard CSE6 as the most appropriate acceleration factor.

Although only the cervical spine was assessed in detail in our study, we have demonstrated the feasibility of a measurement covering the entire spinal cord and its surrounding CSF space in two stacks in 14 min. Such an acquisition allows for a more comprehensive visualization of CSF dynamics and may provide important diagnostic information for patients with syringomyelia, Chiari malformation or arachnoid adhesions [35–37].

Our study has several limitations. The study assessed only a relatively small number of healthy subjects. However, the results of our study clearly show the feasibility of CSE6 accelerated 4D flow MRI of the CSF dynamics at the cervical spinal canal. With a higher number of subjects, we would expect standard deviations and level of agreements to be even smaller. Since no patient data were recorded, these results are not fully transferable to patients with abnormal, more complex flow dynamics. With CSE6, however, we have chosen a rather conservative acceleration factor, so that its use in everyday clinical practice seems conceivable. Quantitative analysis was only provided for the CSF flow at the cervical spinal canal. However, as the feasibility of an imaging volume covering the entire spinal cord was shown in one subject with sufficient image quality, the results are likely to be applicable to whole spine measurements. A specific challenge of whole spine measurements is the correct cardiac gating of both stacks. This could reduce the accuracy of the correct flow dynamics. A major limitation is the missing respiratory gating. Studies by Spijkerman et al. [38] and Yildiz et al. [39] have shown that respiratory gated 2D flow MRI measurements provide more accurate quantifications of peak velocity, net flow, and stroke volume in 2D acquisitions with significant but small differences between respiratory gated acquisitions in inspiration and expiration. However, respiratory gating prolongs the scan time by about a factor of 2 to 3 and thus leads to extremely increased scan times in 4D flow MRI. Therefore, non-respiratory gated 4D flow MRI acquisitions were acquired to achieve clinical feasible scan times. Future studies may enable further acceleration, making respiratory gated 4D flow MRI acquisitions of the spinal CSF feasible with scan times of less than 10 min and thus enable more accurate quantifications of CSF hydrodynamics.

We have chosen a relatively high parallel imaging factor (SENSE) in order to achieve an acquisition with a similar duration as CSE4. A longer SENSE scan with lower acceleration factors would have exceeded the duration of the entire scan protocol. Additionally, although the image quality is increased by lower acceleration factors, the influence of motion artifacts may be more prevalent with increased scan time.

Finally, further sequence optimizations are still applicable. Long reconstruction times can be further improved by application of dedicated reconstruction algorithms [40] and temporal-spatial redundancy may be exploited leading to increased image acceleration [41].

## Conclusions

Our study demonstrates that CSE is a reliable method to accelerate CSF flow measurements. Acceleration factors up to CSE6 seem to be appropriate for clinical use enabling a reduction of acquisition time by 40%. Scan times of about 6 min for the acquisition of a cervical spine imaging volume and about 14 min for a set of two imaging volumes covering the entire spinal cord can be achieved. This facilitates the use of 4D flow MRI for the diagnosis of pathologic CSF dynamics as in Chiari Malformation or syringomyelia.

## Supplementary information

**Additional file 1.** Pathline movie SENSE.

**Additional file 2.** Pathline movie CSE4.

**Additional file 3.** Pathline movie CSE6.

**Additional file 4.** Pathline movie CSE8.

**Additional file 5.** Pathline movie CSE10.

**Additional file 6.** Bland–Altmann plots of absolute net flow.

**Additional file 7.** Bland–Altmann analysis of forward flow.

**Additional file 8.** Bland–Altman analysis of backward flow.

**Additional file 9.** Pathline movie C1-L1.

## Data Availability

The datasets used and/or analyzed during the current study are available from the corresponding author on reasonable request.

## Abbreviations

2D: Two-dimensional
4D: Four-dimensional
AF: Absolute net flow
BF: Backward flow
CI: Confidence interval
CPU: Central processing unit
CS: Compressed sensing
CSE: Compressed SENSE
CSF: Cerebrospinal fluid
ECG: Electrocardiogram
FF: Forward flow
FOV: Field of view
LL: Lower limit
LoA: Limits of agreement
MD: Mean difference
MRI: Magnetic resonance imaging
PCMRI: Phase-contrast magnetic resonance imaging
PV: Peak velocity
REML: Restricted maximum likelihood
ROI: Region-of-interest
SENSE: Sensitivity encoding
SD: Standard deviation
UL: Upper limit
