# Supplementary figures and images for: Compressed-sensing accelerated 4D flow MRI of cerebrospinal fluid dynamics
Source: Fluids Barriers CNS. 2020 Jul 16;17:43. doi: 10.1186/s12987-020-00206-3 (PMC7364783; doi:10.1186/s12987-020-00206-3)

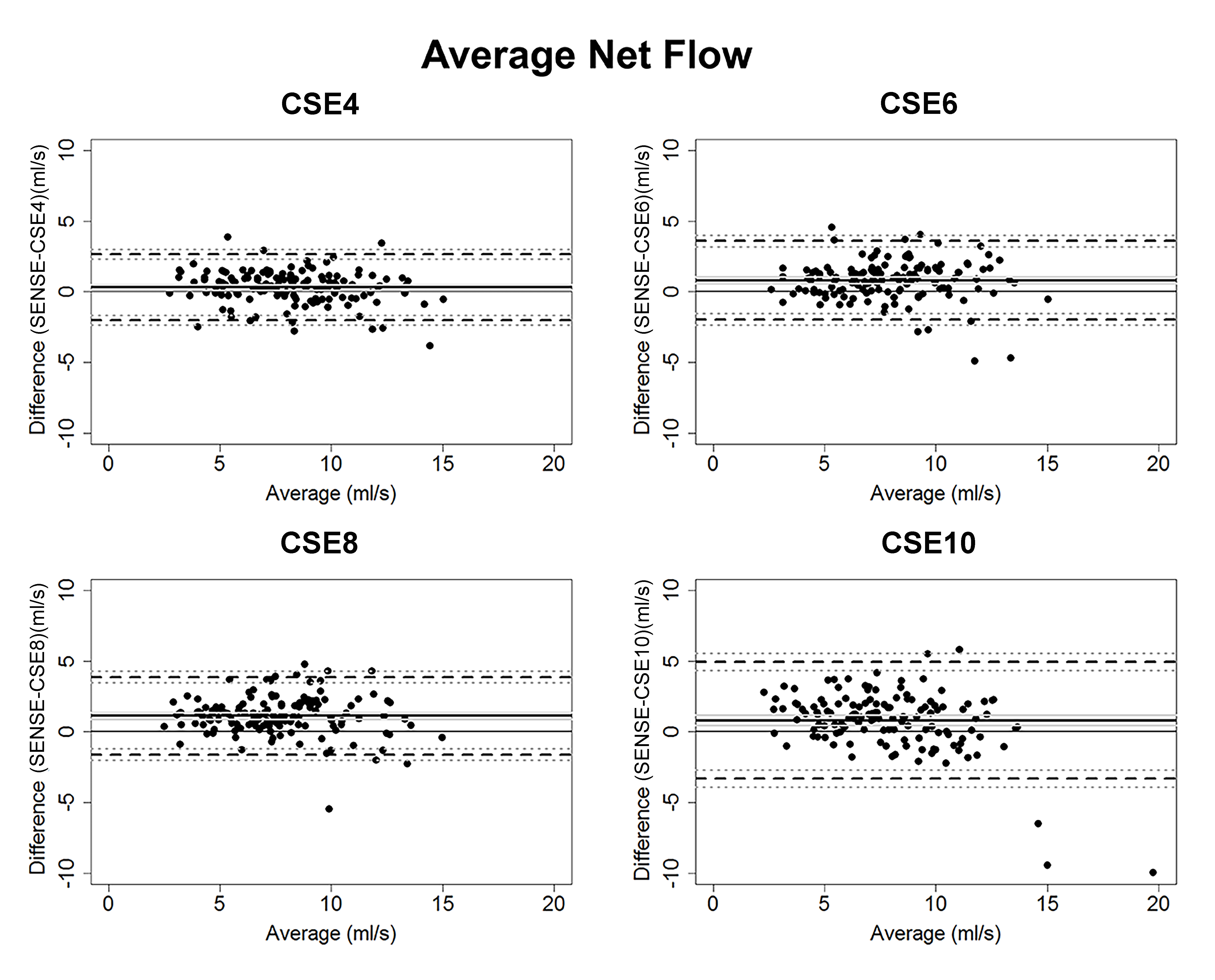

Supplement: Supplementary file 6 — Additional file 6. Bland–Altmann plots of absolute net flow. [file 12987_2020_206_MOESM6_ESM.tiff]

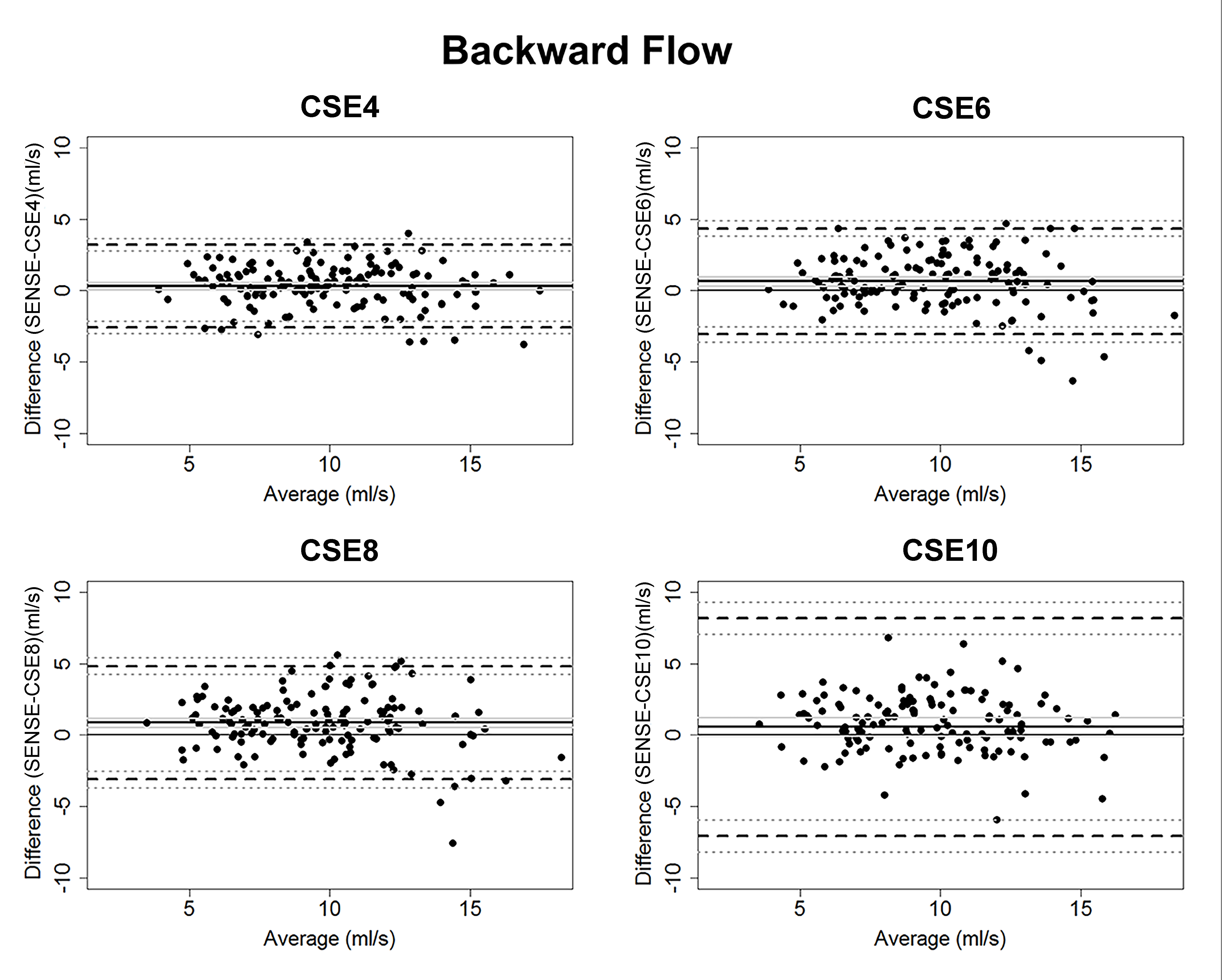

Supplement: Supplementary file 7 — Additional file 7. Bland–Altmann analysis of forward flow. [file 12987_2020_206_MOESM7_ESM.tiff]

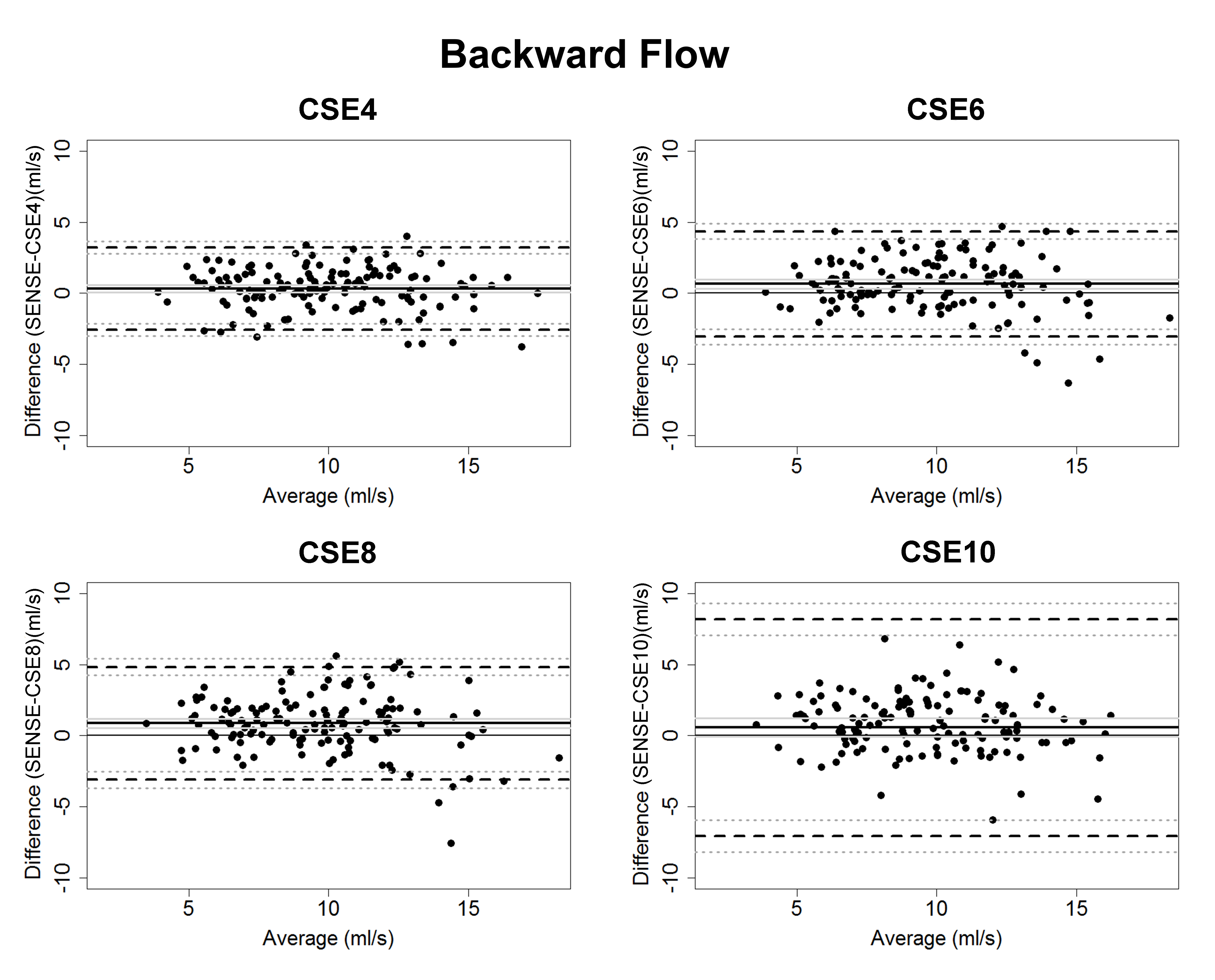

Supplement: Supplementary file 8 — Additional file 8. Bland–Altman analysis of backward flow. [file 12987_2020_206_MOESM8_ESM.tiff]
